# Full-Scale Field Band-Gap-Tuning Test of Mass-Tunable Seismic Metasurface: Design and Validation

**DOI:** 10.3390/ma19214734

**Published:** 2026-11-07

**Authors:** Xinchao Zhang, Changyin Ji, Yulin Lu, Qingfan Shi

**Affiliations:** 1 Key Laboratory of Building Collapse Mechanism and Disaster Prevention, China Earthquake Administration, University of Emergency Management, Langfang 065201, China; zxc@cidp.edu.cn; 2 School of Physics, Beijing Institute of Technology, Beijing 100081, China; jcyinbit@163.com; 3 School of Earthquake Engineering and Building Safety, University of Emergency Management, Langfang 065201, China

**Keywords:** seismic metamaterials, elastic waves, band-gap tuning, energy attenuation, local resonance

## Abstract

**Highlights:**

- Full-scale field tests confirm mass-driven low-frequency band-gap shift on granular-soil sites.
- Wave-field decomposition reveals modal selectivity: BG2/BG3 respond to both Love and Rayleigh waves.
- Mass tuning provides a low-cost, reversible route for in situ site-adaptive band-gap control.

**Abstract:**

Locally resonant seismic metasurfaces can suppress surface waves at sub-wavelength scales, but their fixed configurations are difficult to adapt to site-specific dominant frequency spectra. Seismic surface waves carry large seismic energy and decay slowly with distance, threatening low-rise buildings, lifeline engineering and above-ground infrastructure; site-adaptive surface-wave mitigation is therefore of clear engineering necessity. This paper proposes a mass-tunable spring-resonator seismic metasurface that reconfigures band-gaps in situ by adjusting resonator counterweight mass. Three-dimensional Bloch eigenmode simulations over 2–6 kg show that increasing mass shifts all band-gaps toward lower frequencies, and that at 3 kg or above higher-order modes decouple, generating a fourth band-gap (BG4). Full-scale frequency-sweep tests on a 7 × 6 array on an 8.0 m × 2.0 m × 1.0 m sand bed validate this trend: the measured second band-gap (BG2) shifts from 48–74 Hz at 2 kg to 35–68 Hz at 6 kg. Wavefield decomposition reveals clear modal selectivity: BG2 and the third band-gap (BG3) respond to both Love and Rayleigh waves, whereas BG4 is excited only by Rayleigh waves. Resonator mass is thus a reversible, low-cost, engineering-feasible degree of freedom for in situ band-gap tuning, and wave polarization must be considered in site-adaptive seismic metasurface deployment.

## 1. Introduction

Seismic surface-wave damage represents a critical threat to above-ground civil infrastructure, including low-rise buildings, elevated facilities and lifeline engineering. Surface waves carry substantial seismic energy and decay slowly with propagation distance; therefore, effective mitigation of surface elastic waves remains a long-standing challenge within geotechnical earthquake engineering and vibration-protection communities. Locally resonant seismic metasurfaces belong to a subclass of artificial seismic metamaterials. A seismic metasurface consists of periodic arrays of surface-mounted resonant units placed atop a soil half-space. It leverages local resonance effects to generate sub-wavelength band-gaps, within which elastic surface-wave propagation is significantly suppressed. Different from conventional seismic barriers that rely on Bragg scattering (which requires structural dimensions comparable to seismic wavelengths), locally resonant metasurfaces can achieve wave attenuation at geometric scales much smaller than the target wavelength, breaking the dimensional limitation of traditional isolation measures and offering a promising technical pathway for seismic vibration mitigation.

Since the pioneering proposal of locally resonant phononic crystals, elastic-wave manipulation by artificially engineered periodic resonant media has attracted extensive interdisciplinary research spanning condensed-matter physics, acoustics, civil engineering and geophysics [1]. Liu et al. [2] first demonstrated that resonant structural units can produce sub-wavelength band-gaps, whose underlying physical origin can be interpreted as frequency-dependent negative effective mass density [3]. This unique property matches well the practical demand for low-frequency seismic-wave suppression, motivating abundant investigations on discrete mass-spring resonators and composite metastructure designs [4].

Subsequent research extended locally resonant metamaterial concepts toward field-scale seismic metasurface applications. Brûlé et al. [5] performed field tests and verified that engineered soil configurations are capable of reshaping seismic surface-Wave-fields. Colombi et al. [6] reported that natural forest arrangements function as intrinsic seismic metamaterials, generating Rayleigh-wave band-gaps via tree-trunk local resonance; they further proposed resonant meta-wedge structures to realize mode conversion between Rayleigh surface waves and body waves [7]. Further representative developments include embedded meta-barriers [8], broadband seismic metastructures [9], large-scale seismic metamaterial prototypes [10], inertial resonator arrays [11], and isochronous mechanical-oscillator systems [12], greatly enriching the design library of seismic metamaterials. On the theoretical front, scholars have derived dispersion relations describing coupling effects between surface resonators and Rayleigh waves [13], extended theoretical frameworks for metasurface-based anti-plane Love-wave control [14], and incorporated Biot poroelastic theory to account for fluid–solid coupling within saturated porous foundations [15], which collectively complete the theoretical foundation for half-space soil–resonator interaction problems [16]. Parallel research efforts have covered multi-layer composite layouts, multi-mass resonant cells, periodic foundations and tank-targeted seismic-protection strategies [17,18,19,20,21,22,23]. More recently, nonlinear mechanisms have been introduced to obtain amplitude-dependent dispersion and harmonic-response characteristics for seismic metastructures [24].

Despite the promising wave-attenuation performance demonstrated in existing numerical and laboratory-scale studies, insufficient band-gap tunability constitutes a major bottleneck that hinders real-world engineering deployment of seismic metasurfaces. Actual construction sites exhibit highly variable soil stratification, and the dominant frequency band of incoming seismic surface waves is strongly site-dependent. A metasurface geometry optimized for one specific site may suffer severe performance degradation when deployed under different ground conditions. Several tuning strategies have been reported in the literature to realize reconfigurable band-gaps: modifying boundary constraints for ultra-low-frequency stop-band generation [25], spatially grading resonator heights to alter dispersion landscapes [26], point-wise modulation of the refractive index for elastic metalens construction [27], and exploiting nonlinear stiffness to achieve amplitude-sensitive band-gap shifting [28]. Numerical simulations have also explored mass-in-mass periodic barriers for strong-motion seismic mitigation [29]. Among these competing technical routes, adjusting the resonator lumped-mass stands out with distinct practical merits: the principle is physically intuitive, implementation on-site is straightforward, and the reconfiguration process is fully reversible. By simply adding or removing counterweight materials, one can continuously adjust the natural resonant frequency of each unit cell and thus reconfigure the metasurface band-gap range, which yields attractive application prospects for site-adaptive seismic protection.

Nevertheless, experimental evidence for mass-tunable seismic metasurfaces remains scarce in the published literature. Most existing experimental investigations are limited to small-scale laboratory bench-top tests under ideal boundary conditions [30,31,32]. The limited number of reported field-scale experiments either adopt natural resonant scatterers such as trees [33], or adopt fixed-geometry embedded rigid structures without on-site tunable capability [5,8]. To our knowledge, full-scale field experiments using artificial spring-mass seismic metasurfaces with in situ adjustable counterweights deployed on real granular-soil sites—which additionally enable quantitative decomposition of Love-wave and Rayleigh-wave attenuation and multi-parameter cross-validation against Bloch-theoretic numerical simulations—have rarely been documented.

To fill this research gap, the present work selects resonator mass as the core tuning variable and systematically investigates its modulation effects on multi-order locally resonant band-gaps. First, three-dimensional Bloch eigenmode simulations are performed over a mass range of 2–6 kg to reveal the physical mechanisms through which mass variation drives band-gap downward-frequency shifts and higher-order-mode decoupling that generates new band-gaps. Following the numerical parametric scan, full-scale frequency-sweeping field experiments are conducted on an 8.0 m × 2.0 m × 1.0 m sand-filled foundation equipped with a 7 × 6 resonator array. We quantitatively evaluate total-energy attenuation as well as separated attenuation contributions from Love waves and Rayleigh waves, and compare measured responses against simulation predictions. Adopting a research paradigm combining “parametric numerical scanning + full-scale field validation at representative mass limits”, this study verifies that resonator mass serves as an effective degree-of-freedom for band-gap tuning. It provides combined theoretical and experimental support for the future engineering design of site-adaptive seismic metasurfaces. Note that the term “full-scale” in this manuscript indicates prototype-sized resonators deployed on real soil sites, rather than implying that ultra-low-frequency seismic band-gaps are achieved.

## 2. Theoretical Modeling and Numerical Simulation

To interpret the mass-tunable band-gap physics observed in subsequent field experiments, this chapter establishes the theoretical and numerical basis for the investigated seismic metasurface. We construct a dynamic resonator model that accounts for multi-modal motion and mass-sensitive soil–structure contact, and perform Bloch eigenmode simulations to quantify how resonator mass reshapes band-gap features. These numerical predictions will later serve as a baseline reference for comparison against full-scale experimental measurements presented in Section 3.

### 2.1. Multi-Degree-of-Freedom Resonator Model and Mass-Tuning Principle

**Figure 1:**
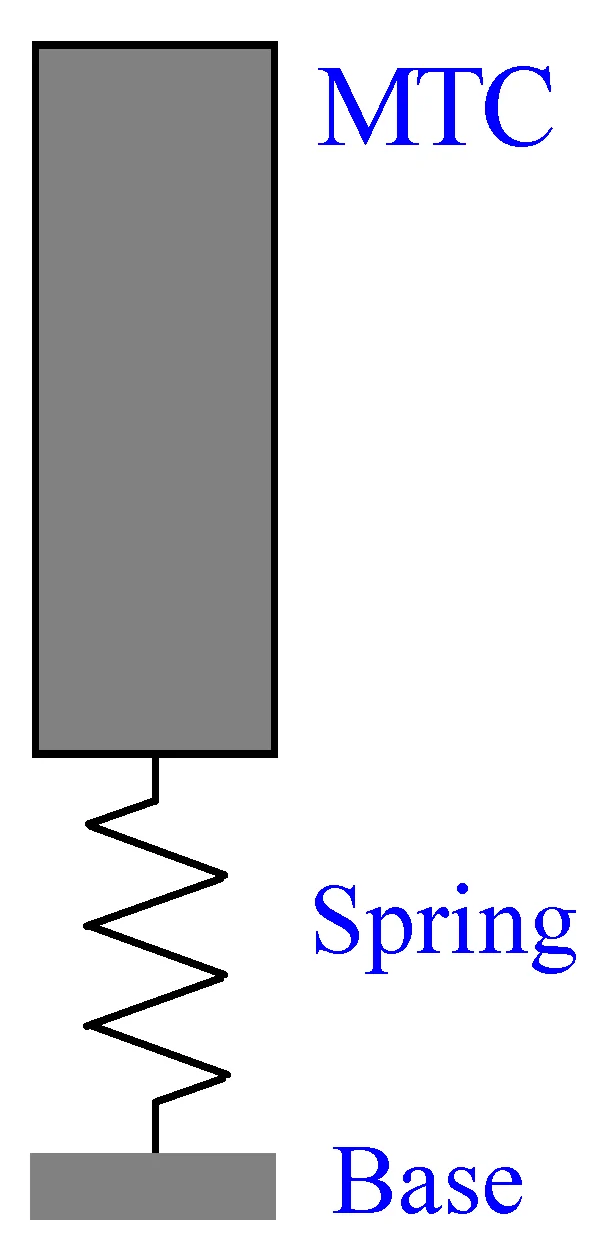
Spring–oscillator model.

According to the existing literature, each unit cell of surface-mounted seismic metamaterials can be simplified to a spring-mass dynamic assembly [2,34], as illustrated in Figure 1. The device consists of a mechanical spring, a mass-tuning cup (MTC) for variable counterweight filling, and a wooden base plate in contact with the sandy substrate. Its detailed dimensions are given in Section 2.2.

Unlike an ideal single-degree-of-freedom lumped-mass oscillator, the real resonator unit can undergo both translational and rotational motions. Accordingly, each resonator unit cell is idealized as a rigid body with six degrees of freedom (six-DOF), possessing three translational DOFs along *x*, *y*, *z* directions (*u_x_*, *u_y_*, *u_z_*) and three rotational DOFs (*θ_x_*,*θ_y_*,*θ_z_*) around these axes [16,35]. This multi-DOF model corresponds physically to our real prototype: translational modes represent horizontal/vertical shaking of the filled cup-base assembly; rotational modes represent rocking and torsional deformation observed in physical tests.

The generalized coordinate vector for this rigid-body unit-cell resonator is defined as:

(1) $$q={\left[u_{x},u_{y},u_{z},\theta_{x},\theta_{y},\theta_{z}\right]}^{T}$$

where *q* is the generalized displacement vector (three translational plus three rotational generalized coordinates). The dynamic governing equation of the unit cell is then expressed as:

(2) $$M\overset{\cdot \cdot }{q}+C\overset{\cdot }{q}+[k_{s}+k_{c}(m)]q=F_{\mathrm{wave}}$$

where *M* = diag(*m*, *m*, *m*, *I_x_*, *I_y_*, *I_z_*) denotes the mass matrix, in which mass *m* is treated as a tunable structural parameter; *I_x_*, *I_y_*, and *I_z_* are the inertia about the three orthogonal axes; *C* denotes the damping coefficient; *k*_s_ represents the internal stiffness contributed by springs, cup components, and connecting structures; *k*_c_(*m*) is the contact stiffness between the base plate and sand substrate, which varies with structural mass rather than remaining constant; and *F*_wave_ denotes the generalized forces and moments imposed by substrate surface waves at the contact interface.

To characterize the mass-dependent contact behavior, a power-law empirical stiffness model is adopted [36]:

(3) $$k_{c}(m)=k_{c0}+k_{c1}{\left(m\right)}^{p}$$

where *k*_c0_ is the initial contact stiffness under zero preload, *k*_c1_ is the fitting coefficient, and *p* is the power-law exponent typically ranging from 0.5 to 1. Physically, an increase in the lumped-mass enhances the normal pressure exerted by the base plate on the sand surface, which further alters the interfacial contact stiffness. Accordingly, the modal characteristics of the resonator can be effectively tuned by adjusting the structural mass.

Based on the Rayleigh quotient principle, the *w_n_* order natural angular frequency of the system is formulated as:

(4) $$\omega_{n}^{2}(m)=\frac{\varphi_{n}^{T}[k_{s}+k_{c}(m)]\varphi_{n}}{\varphi_{n}^{T}M\varphi_{n}}$$

where Φ*_n_* represents the *n* modal shape vector of the unit-cell system. It should be noted that the eigenvector Φ*_n_* in the Rayleigh quotient corresponds to the generalized modal vector of the lumped-parameter six-DOF rigid resonator. The periodically arranged metamaterial excites local resonance through harmonic vibration at the surface; the out-of-phase coupling between the resonators and the substrate generates negative effective dynamic parameters [2,34,37], thereby dissipating seismic wave energy and forming band-gaps. Thus, altering the surface mass enables tuning of the natural frequency and active band-gap control.

### 2.2. Unit-Cell Geometry and Material Parameters

**Figure 2:**
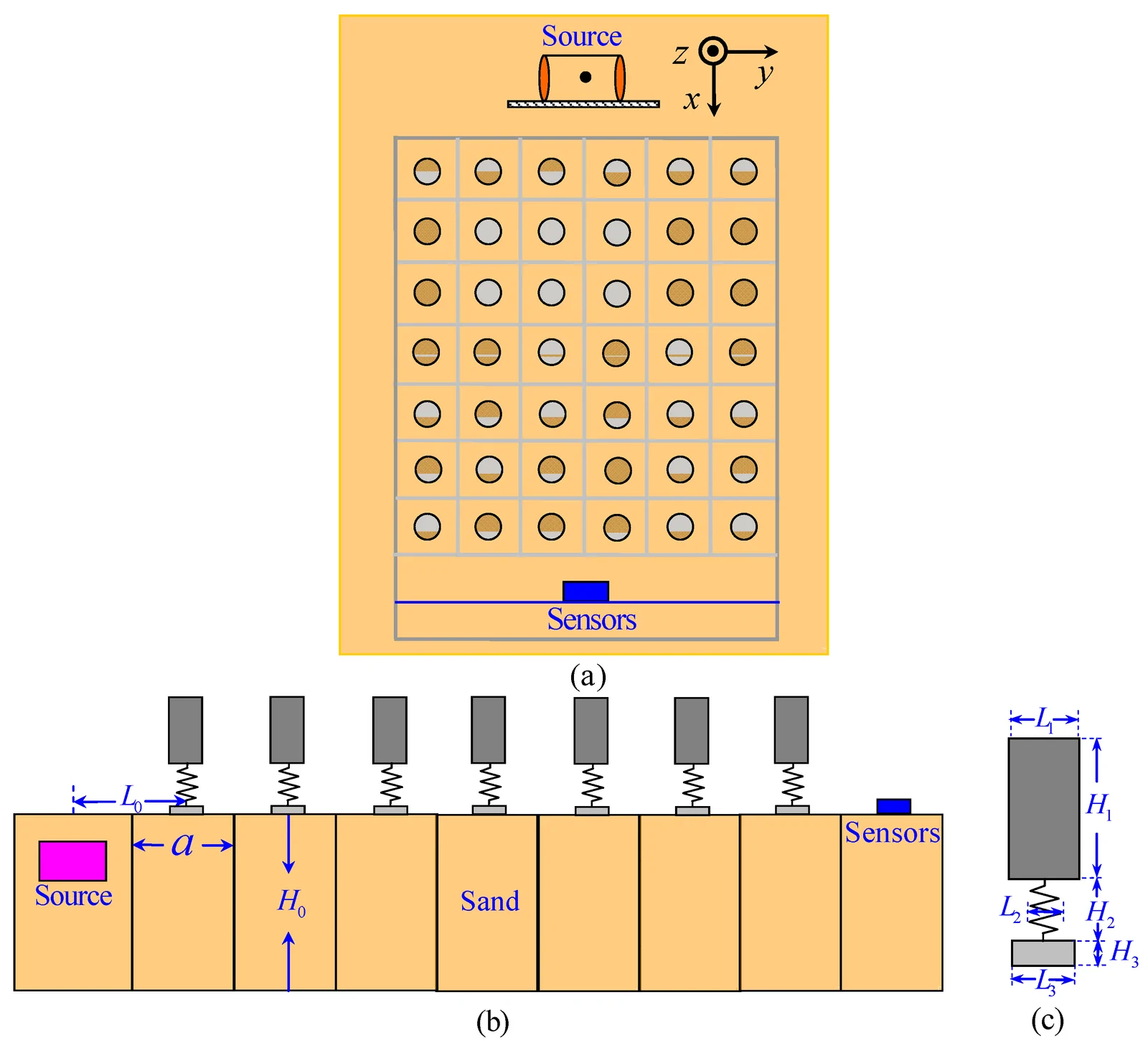
Design schematics: (**a**) plan view, (**b**) cross-sectional view, (**c**) spring-resonator configuration.

Figure 2 presents the plan view (a), cross-sectional view (b), and schematic diagram of the spring–oscillator unit (c) for the proposed structure. The lattice constant *a* is 30 cm. The spring adopted is a standard manganese-steel die spring with a free height of 50 mm, outer diameter of 40 mm, inner diameter of approximately 20 mm, maximum compression ratio of 40%, and axial stiffness of 1.059 × 10^5^ N/m. The base is a poplar plate with dimensions of 60 mm × 60 mm × 10 mm. The oscillator consists of a cylindrical polyethylene terephthalate (PET) cup with a diameter of 100 mm, height of 200 mm, and wall thickness of 2 mm. Detailed design parameters are summarized in Table 1 and Table 2. The spring is rigidly bolted to both the PET cup and the poplar base plate. The ballast mass (2, 3, 4, 5, and 6 kg) serves as the sole tuning parameter in this study. The masses of 2 kg and 6 kg are achieved by filling the cup with river sand and iron sand up to a height of 0.20 m, respectively, whereas masses ranging from 3 to 5 kg are obtained by mixing river sand and iron sand at different proportions.

The geometric dimensions are listed in Table 1. The material parameters of the designed unit cell are listed in Table 2, in which the parameters were determined from field measurements and engineering handbooks.

### 2.3. Numerical Results: Band-Gap Evolution and Modal Responses

Based on the propagation wave equation of elastic waves and Bloch’s theorem [38,39], we obtain the eigenvalue equation for periodic structures.

(5) $$[D(k)-\omega^{2}M]u(r,t)=0$$

where ***D***(***k***) is the stiffness matrix, ***M*** is the mass matrix, *ω* is the angular frequency, ***k*** is the wave vector, and ***u***(***r***,*t*) is the displacement field, which is a function of the position vector ***r*** and time *t*. Based on this formulation, finite element simulations are conducted using COMSOL Multiphysics (5.2, COMSOL AB, Stockholm, Sweden). 

The dispersion relations, ω(k), are computed by sampling wave-vector points along the irreducible Brillouin-zone high-symmetry path M–Γ–X–M. This path covers the critical high-symmetry directions within the first Brillouin zone of the square lattice. By scanning wave vectors along these directions, we obtain the upper- and lower-frequency bounds of complete band-gaps for surface-wave propagation in the 2D periodic metasurface. Band-gap intervals are identified where no real propagating eigensolutions exist. Using these dispersion relations, the band-gap characteristics for different structural parameters can be quantitatively compared.

#### 2.3.1. Modal Characteristics Under Representative Mass Conditions

**Figure 3:**
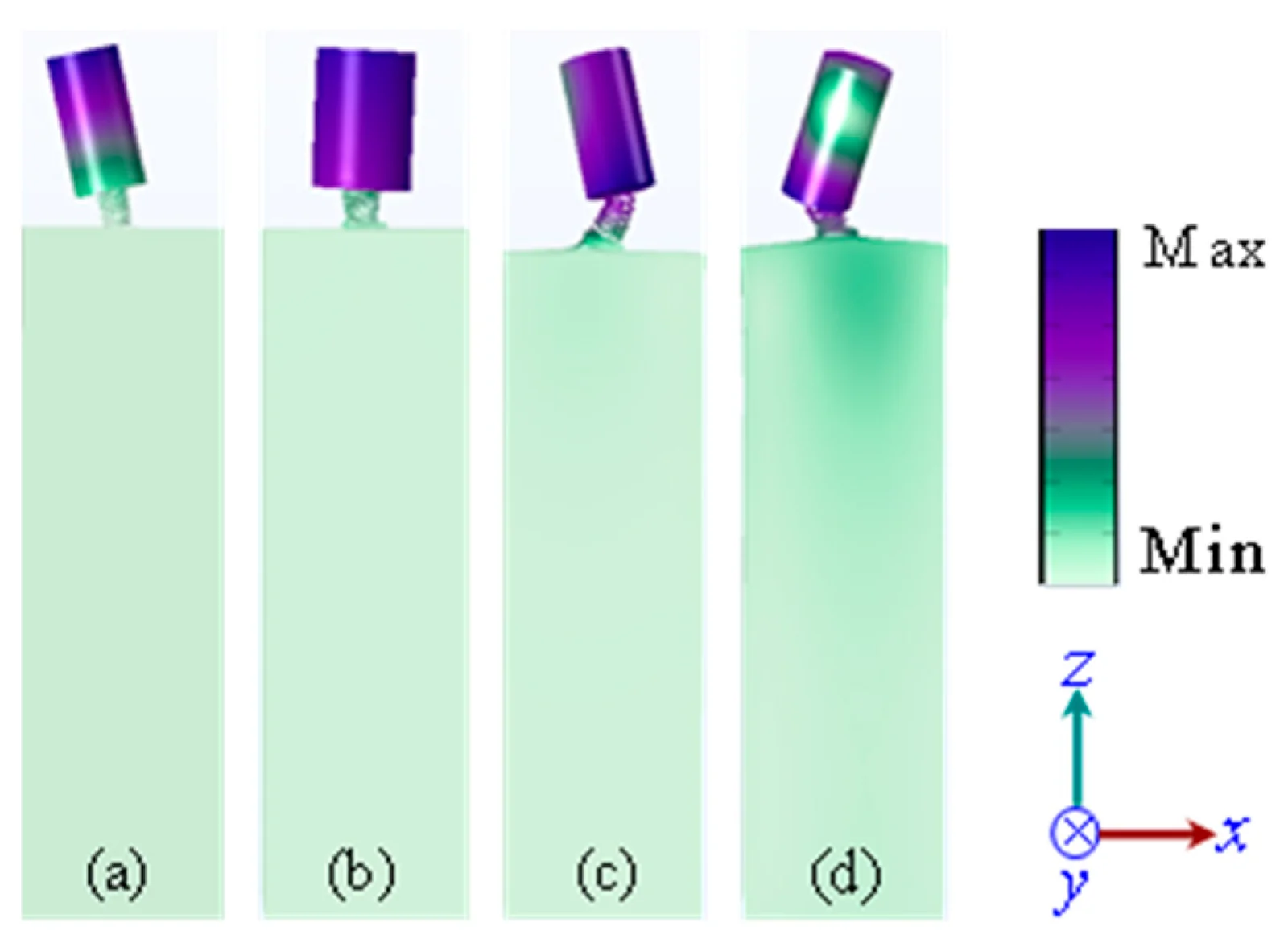
Eigenmode shapes of the 2 kg resonator: (**a**) *f*_x_ = 8.8 Hz (horizontal translation); (**b**) *f*_m_ = 48.3 Hz (mixed translation–rocking); (**c**) *f*_r1_ = 75.1 Hz (rocking); (**d**) *f*_r2_ = 109.6 Hz (higher-order rocking/shear–tilt).

**Figure 4:**
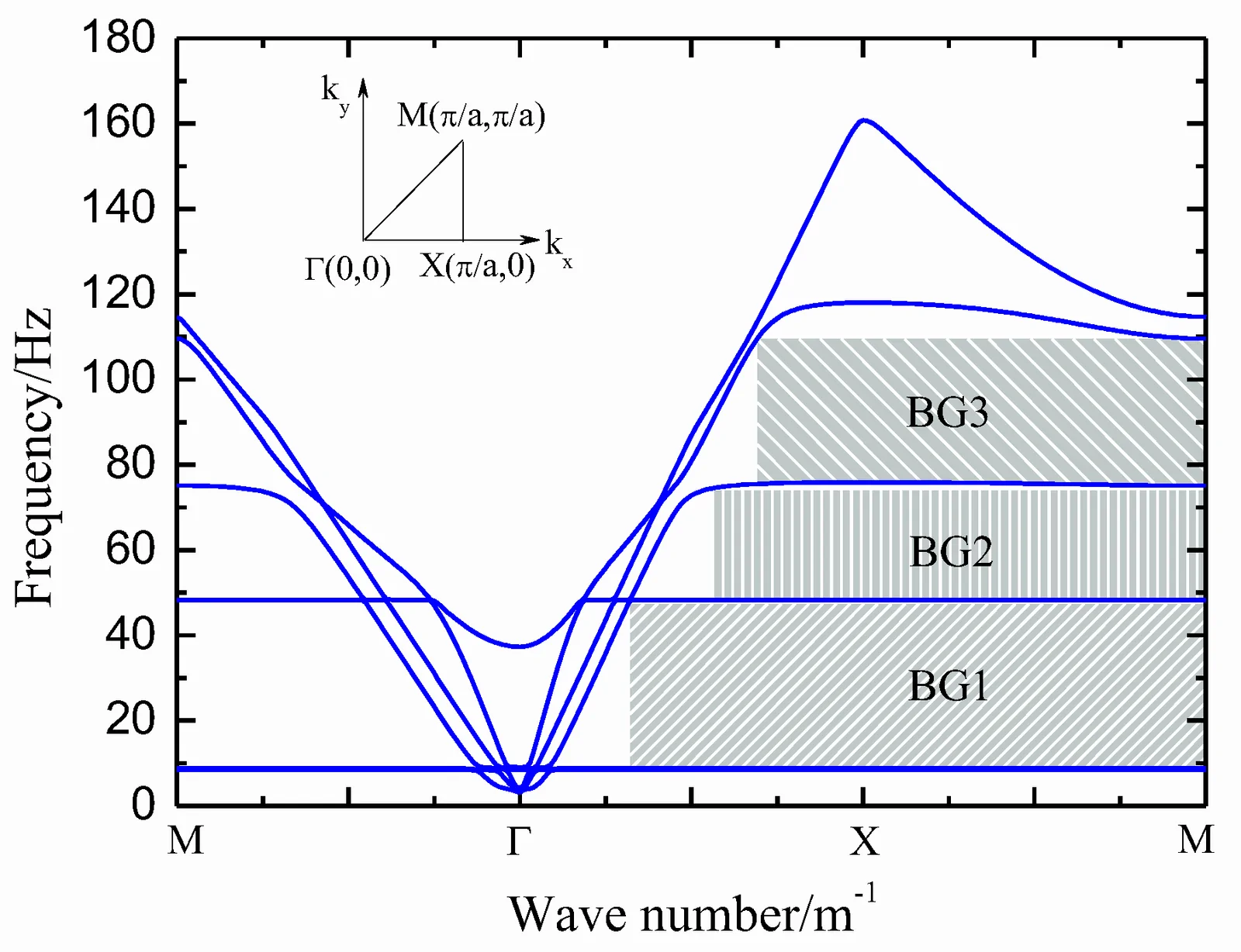
Band-gap diagram for the 2 kg resonator, showing three complete band-gaps (BG1–BG3).

**Figure 5:**
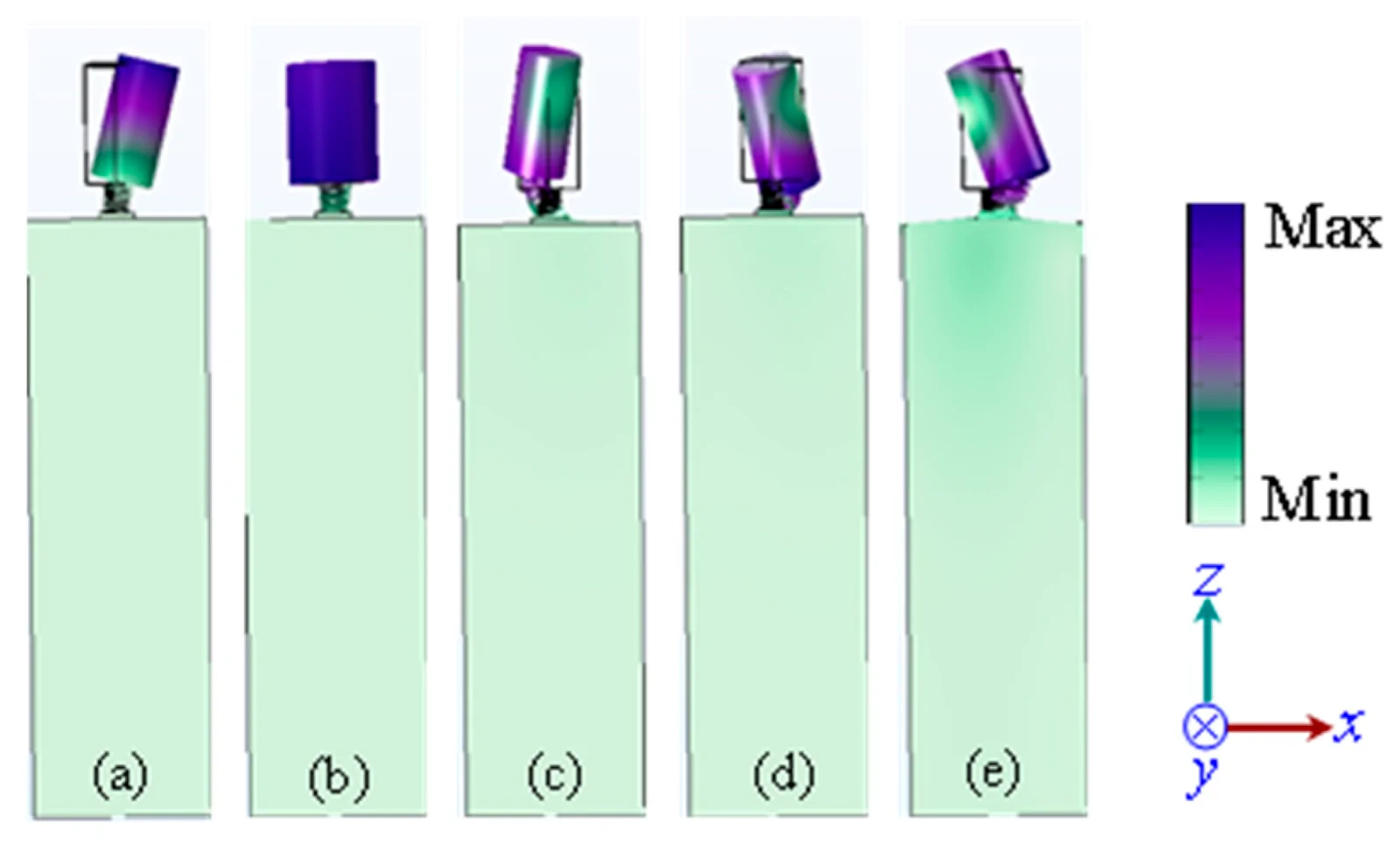
Eigenmode shapes of the 6 kg resonator: (**a**) *f*_x_ = 7.7 Hz (horizontal translation), (**b**) *f*_m_ = 35.8 Hz (mixed translation–rocking), (**c**) *f*_r1_ = 66.9 Hz (rocking), (**d**) *f*_r2_ = 87.2 Hz (higher-order rocking/shear–tilt), (**e**) *f*_r3_ =103.7 Hz (out-of-plane torsional).

**Figure 6:**
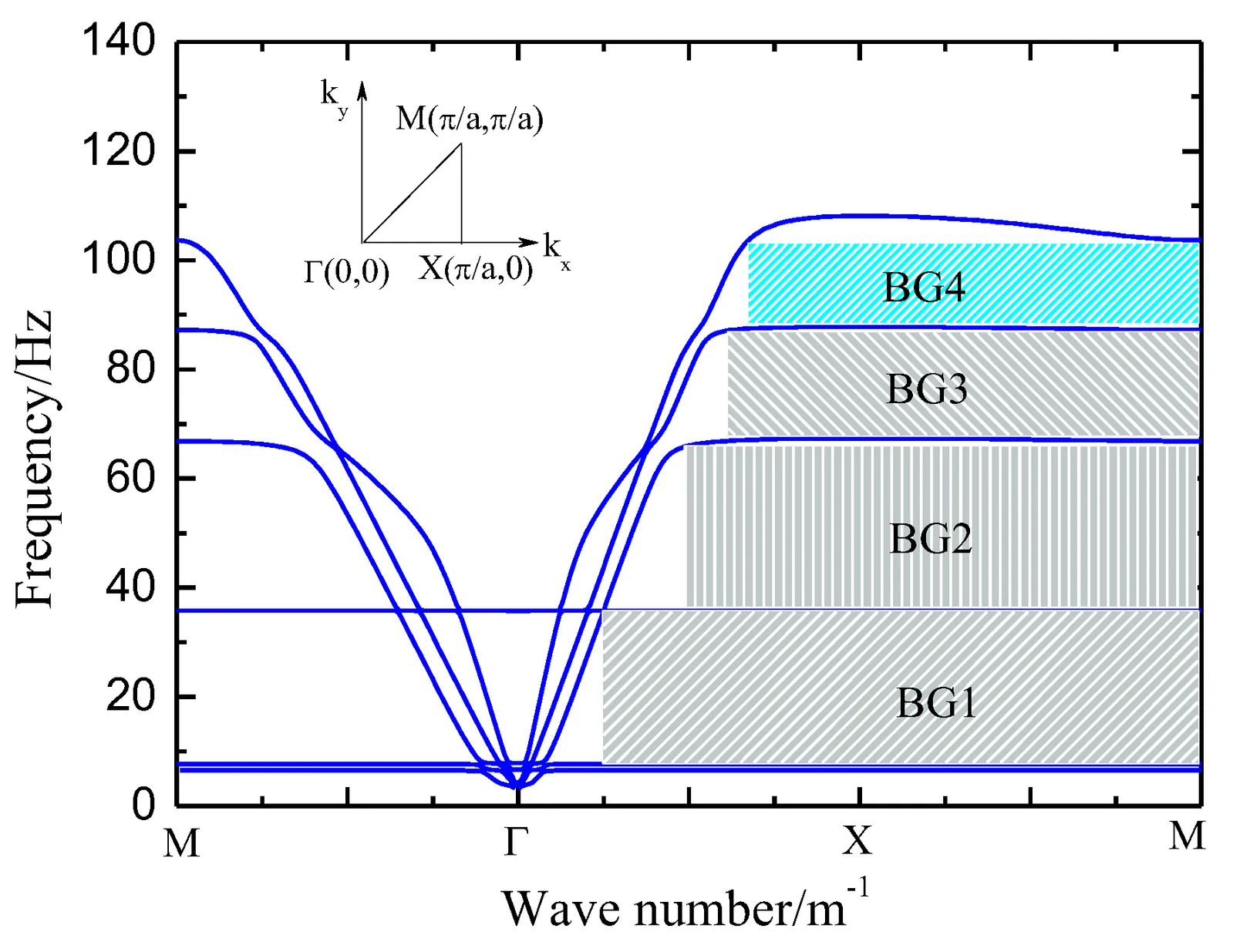
Band-gap diagram for the 6 kg resonator, showing four complete band-gaps (BG1–BG4).

Guided by the six-DOF rigid-body resonator model established in Section 2.1 (Equations (1)–(4)), three-dimensional Bloch eigenmode analyses were performed for the two representative masses that corresponding directly to the experimental configurations, namely 2 kg (river-sand filling) and 6 kg (iron-sand filling). Figure 3 and Figure 4 display the computed eigenmode shapes and corresponding band structures of the 2 kg resonator, respectively, while Figure 5 and Figure 6 display those of the 6 kg resonator. Within the six-DOF framework, each eigenmode can be projected onto the generalized coordinate vector *q* = [*u*_x_, *u*_y_, *u*_z_, *θ_x_*, *θ_y_*, *θ_z_*]ᵀ, so that its dominant translational or rotational content can be identified unambiguously.

As shown in Figure 3, the 2 kg resonator exhibits four identifiable modes: the horizontal translation *f*_x_ = 8.8 Hz, the mixed mode *f*_m_ = 48.3 Hz, and two rotational modes *f*_r1_ = 75.1 Hz and *f*_r2_ = 109.6 Hz. The latter three open BG1, BG2, and BG3, respectively (Figure 4). The horizontal mode *f*_x_ does not open an independent gap: being dominated by the lateral spring stiffness, the resonator moves almost in phase with the substrate surface motion at the wavelengths of interest, so the out-of-phase coupling is too weak to drive the effective dynamic parameters negative over a finite frequency interval; the mode therefore manifests only as a resonance adjacent to the passband rather than a complete band-gap.

As shown in Figure 5, the 6 kg resonator exhibits five modes—*f*_x_ = 7.7 Hz (horizontal translation), *f*_m_ = 35.8 Hz (mixed), *f*_r1_ = 66.9 Hz, *f*_r2_ = 87.2 Hz, and *f*_r3_ = 103.7 Hz (the latter three rotational modes)—which collectively open four complete band-gaps, BG1–BG4 (Figure 6). A clear one-to-one correspondence exists between the modal eigenfrequencies and the upper band-gap edges (2 kg: 47.5, 74.4, and 109.3 Hz versus *f*_m_, *f*_r1_, and *f*_r2_; 6 kg: 35.7, 65.8, 87.6, and 103.1 Hz versus *f*_m_, *f*_r1_, *f*_r2_, and *f*_r3_), confirming the multi-order locally resonant origin of the band-gaps. Whenever the incident surface-wave frequency approaches an eigenfrequency of the resonator, out-of-phase coupling between the resonator and the substrate drives the effective dynamic parameters toward negative values; elastic traveling waves can then no longer propagate within that frequency range, and a band-gap forms. Each decoupled higher-order mode contributes one additional attenuation band. 

The mass-driven frequency downshift follows directly from the Rayleigh quotient in Equation (4). Increasing the ballast mass *m* enlarges both the translational mass *m* and the mass moments of inertia *I*_x_, *I*_y_, and *I*_z_ in the mass matrix ***M***, whereas the mass-dependent part of the generalized stiffness stems solely from the baseplate–sand contact stiffness *k*_c_(m) = *k*_c0_ + *k*_c1_(*m*)*^p^* (Equation (3)), which grows only sublinearly (0.5 ≤ *p* ≤ 1), while the internal spring stiffness *k*_s_ remains unchanged. The denominator of Equation (4) therefore grows faster than the numerator, and all locally resonant modal frequencies decrease with increasing mass (*f*_m_: 48.3 → 35.8 Hz; *f*_r1_: 75.1 → 66.9 Hz; *f*_r2_: 109.6 → 87.2 Hz). Consequently, BG1–BG3 all shift toward lower frequencies along M–Γ–X–M; in particular, the upper edge of BG1 decreases from approximately 47.5 Hz to approximately 35.7 Hz, which markedly enhances the low-frequency suppression capability—the most valuable improvement for seismic locally resonant metasurfaces. Meanwhile, the low-frequency acoustic branches, corresponding to the collective motion of the unit cell together with the substrate, are governed primarily by the soil parameters and remain almost unchanged, whereas the locally resonant optical-like branches shift downward. 

The emergence of BG4 originates from the decoupling of higher-order modes. At a mass of 2 kg, *f*_r2_ and *f*_r3_ are closely spaced and strongly hybridized, so the corresponding optical-like branches remain connected and no complete gap exists above BG3. As the mass increases, the rotational inertia grows in proportion to *m*, while *k*_c_(*m*) increases only sublinearly; modes with different rotational polarizations therefore possess different stiffness-to-inertia ratios and downshift at different rates. The modal spacing progressively widens, the hybridization weakens and eventually vanishes, and the previously near-degenerate *f*_r3_ separates out, opening the fourth complete band-gap BG4 from a mass of 3 kg onward (Table 3; 88.8–103.1 Hz at 6 kg). Because the generalized stiffness of the rocking and torsional modes contains a significant contribution from the contact stiffness *k*_c_(*m*), the higher-order band-gaps BG3 and BG4 are intrinsically sensitive to in situ contact conditions—a property that will reappear in the simulation–experiment comparison presented in Section 4. 

Finally, the six-DOF model naturally predicts the modal selectivity of the band-gaps. Within the experimentally accessible frequency range (Section 4), the rocking/shear–tilt modes *f*_r1_ and *f*_r2_ (BG2 and BG3) can be excited by both horizontally polarized Love waves and Rayleigh waves, whereas excitation of the out-of-plane torsional mode *f*_r3_ requires a vertical input and can therefore only be triggered by Rayleigh waves possessing vertical vibration components. BG4 is thus expected to appear only in the Rayleigh-wave attenuation spectrum—a prediction verified by the full-scale field tests in Section 4. It should also be noted that increasing the mass lowers the resonance frequencies at the cost of additional structural self-weight; a trade-off between low-frequency performance and structural weight is therefore required in practical engineering design. 

The identified modes and their correspondence to the band-gaps are summarized in the Table 3.

#### 2.3.2. Parametric Analysis of Band-Gap Variation

Therefore, with other parameters held constant, the band-gaps for resonator masses ranging from 2 to 6 kg (achieved by different river sand/iron sand ratios) were computed via simulation, with results summarized in Table 4.

The numerical results demonstrate that increasing the resonator mass shifts all band-gaps toward lower frequencies, which is beneficial for low-frequency seismic wave control; however, the bandwidth of the first band-gap narrows accordingly. When the mass reaches 3 kg or above, higher-order locally resonant modes are excited, generating the fourth band-gap and further extending the vibration suppression frequency coverage. The responses of different band-gap orders to mass variation differ significantly; simply increasing the resonator mass cannot simultaneously optimize the low-frequency onset and broadband performance across all orders. Therefore, practical engineering design must comprehensively consider the onset frequency, bandwidth, and band-gap order to rationally optimize the resonator mass.

## 3. Full-Scale Field Experiment

### 3.1. Experimental Setup and Test Conditions

**Figure 7:**
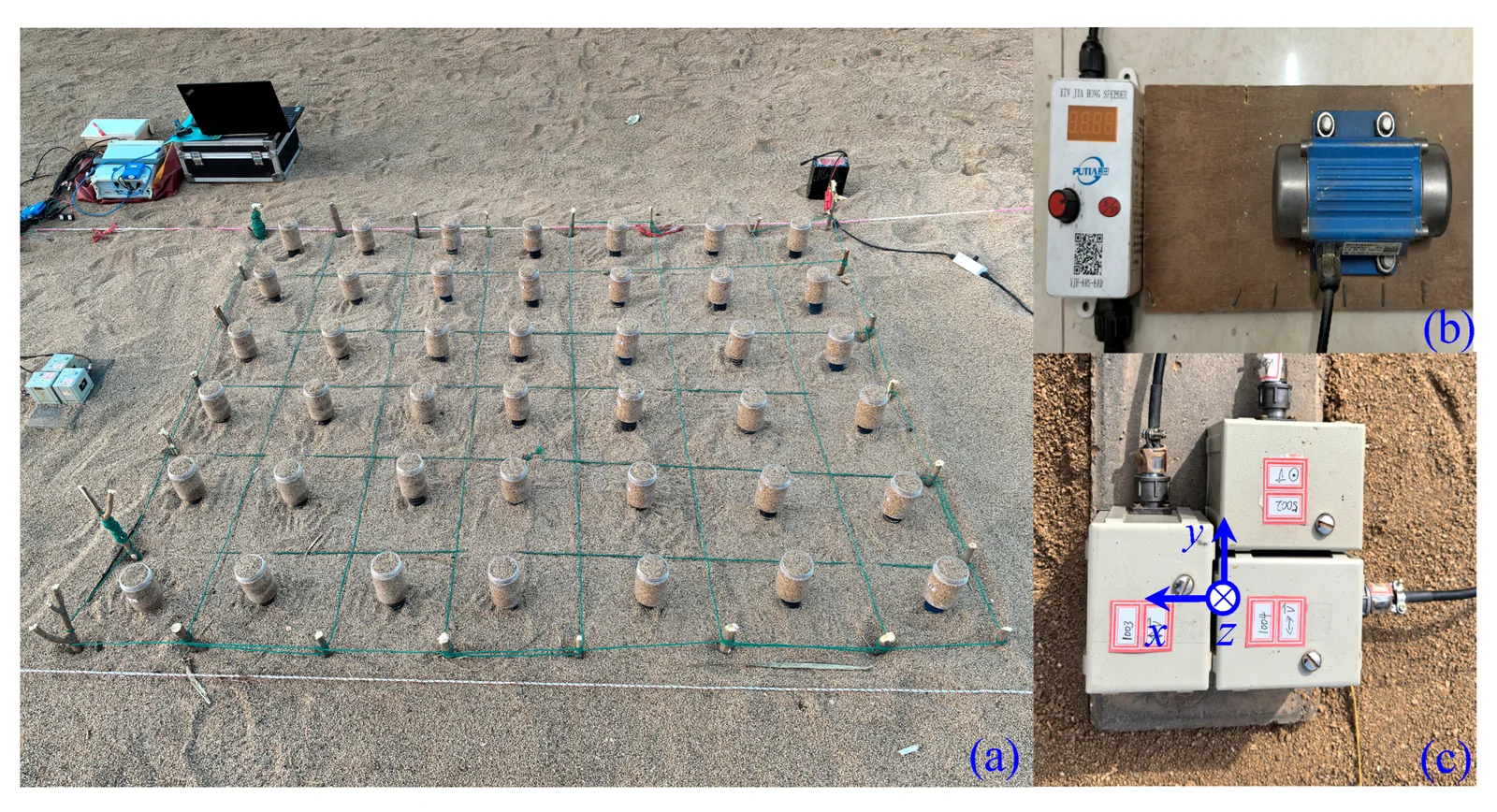
Photographs of the experimental site: (**a**) site overview, (**b**) vibration source, (**c**) sensors.

A full-scale field experiment was conducted using river sand with a particle size of 1–3 mm, piled into a sand bed of approximately 8.0 m × 2.0 m × 1.0 m (length × width × height). A 7 × 6 array of resonators was arranged on the sand bed according to the unit cell configuration shown in Figure 7a. The ambient temperature during the experiment ranged from 20 to 25 °C, and the site remained dry.

A variable-speed eccentric rotating mass shaker was used as the vibration source, with a maximum excitation force of 1 kN and continuously tunable frequency from 20 to 130 Hz, as shown in Figure 7b. The shaker was buried approximately 50 cm deep in the sand bed—equivalent to a buried harmonic source in the Lamb problem sense [40]. Considering that excitation energy at very low frequencies would be indistinguishable from background noise, the frequency was swept from 21 Hz to 123 Hz at 3 Hz intervals, with a frequency error of approximately ±0.5 Hz. As defined in Figure 2, the horizontal axial direction is denoted as the x-direction, the horizontal transverse direction as the y-direction, and the vertical direction as the z-direction. A three-component sensor (bandwidth 0.5–150 Hz, low-frequency cut-off −3 dB) was placed 0.5 m from the array center at the front-facing position to record data, as shown in Figure 7c. The sensor and the 24-bit data acquisition system were powered by 12 V and 24 V DC batteries, respectively. The resonators were designed with detachable containers for rapid mass replacement.

### 3.2. Data Processing and Surface-Wave Decomposition

The experimental procedure consisted of: (1) measuring background energy, (2) measuring the energy under no-structure conditions at different excitation frequencies, and (3) measuring the energy distribution with the surface resonators installed. The excitation frequency of the eccentric shaker was adjusted; considering the nonlinear effects among sand particles, data were considered valid only when the dominant frequency recorded by the sensor matched the shaker frequency. The average energy per unit time was calculated by numerical integration, and energy exceeding five times the background level was regarded as effective.

The recording duration was set to 120 s, with the stable middle interval from *t*_1_ = 40 s to *t*_2_ = 80 s selected for analysis. The average energy *E_i_* per unit time in each direction was calculated as:

(6) $$E_{i}=\frac{1}{t_{2}-t_{1}}\int_{t_{1}}^{t_{2}}\frac{1}{2}m_{i}v_{i}^{2}dt\;(i=x,y,z)$$

where *m_i_* is the sensor mass and *v_i_* is the instantaneous velocity recorded in each direction. The energy attenuation characteristics were then computed by comparing the energy at different frequency components at the same measurement point. The energy without surface resonators (no-structure condition) is denoted as *E*_0_, and the energy with resonators as *E*_s_, with corresponding components *E*_0*i*_ and *E*_s*i*_ (*i = x*,*y*,*z*).

(7) $$\left\{\begin{array}{l} E_{0}=\sum_{i=1}^{3}E_{0i}\\ E_{s}=\sum_{i=1}^{3}E_{si} \end{array}\right.(i=x,y,z)$$

The total-energy attenuation coefficient *k*_s_ and component energy attenuation coefficients *k*_s*i*_ are defined as:

(8) $$\left\{\begin{array}{l} k_{s}=10\times \mathrm{lg}(\frac{E_{s}}{E_{0}})\\ k_{si}=10\times \mathrm{lg}(\frac{E_{si}}{E_{0i}})\;(i=x,y,z) \end{array}\right.$$

Considering that Love waves result from the superposition of horizontally polarized shear waves (SH) after multiple reflections from resonators, they are contained in the *y*-direction measurements [41,42]; Rayleigh waves result from the superposition of horizontal progressive waves (P) and vertical shear waves (SV) through the resonators, and are thus contained in the sum of *x*- and *z*-direction measurements [40,43,44]. The Love- and Rayleigh-wave attenuation coefficients, *k*_L_ and *k*_R_, are accordingly defined as:

(9) $$\left\{\begin{array}{l} k_{L}=10\times \mathrm{lg}(\frac{E_{sy}}{E_{0y}})\\ k_{R}=10\times \mathrm{lg}(\frac{E_{sx}+E_{sz}}{E_{0x}+E_{0z}}) \end{array}\right.$$

## 4. Results and Discussion

### 4.1. Total-Energy Attenuation Performance

**Figure 8:**
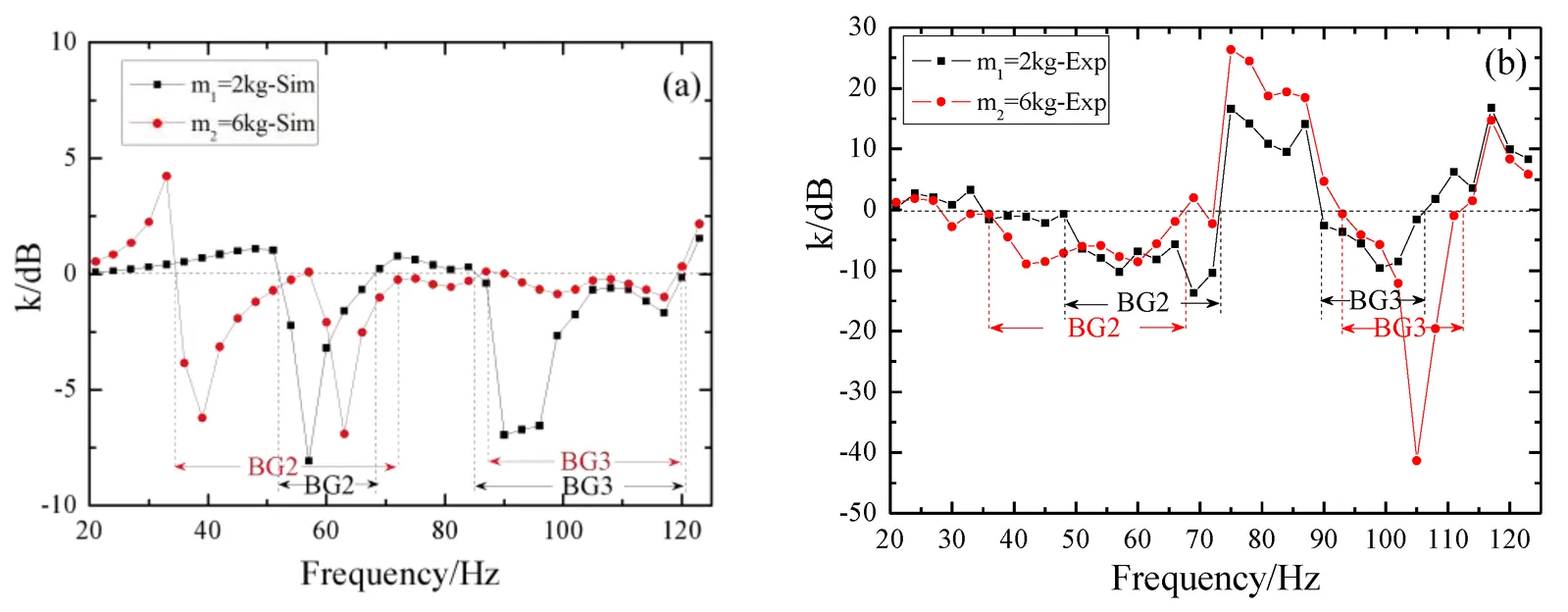
Total-energy attenuation: (**a**) simulation results, (**b**) experimental results.

Figure 8 presents the simulated and full-field measured results for total-energy attenuation. To verify the reliability of the experimental results, the simulations in this section are based on the actual experimental structure shown in Figure 7. Using Equation (8), the transmission coefficients of the resonators with masses of 2 kg and 6 kg are calculated separately to analyze the variation in the band-gaps.

Owing to experimental limitations, the low-frequency BG1 is difficult to observe in the tests; therefore, we begin our discussion with BG2. In terms of total-energy attenuation, the simulation results show that for the 2 kg resonator, the main attenuation band, BG2, is located at approximately 48–73 Hz. When the mass is increased to 6 kg, BG2 shifts downward to approximately 35–72 Hz, with decreases of about 13 Hz and 1 Hz at the lower and upper boundaries, respectively, indicating a clear mass-driven low-frequency shift trend. The measured results are highly consistent with this trend: the measured attenuation intervals are approximately 48–74 Hz for the 2 kg resonator and 35–68 Hz for the 6 kg resonator. As the mass increases, the attenuation band overall shifts toward lower frequencies, and the suppression capability at the low-frequency end is enhanced. This indicates that using the resonator mass as a tunable degree of freedom can indeed provide control over the position of the attenuation band under actual site conditions, and the tuning direction agrees with the simulation predictions based on actual parameters.

In the high-frequency range (BG3), the simulated attenuation intervals are 85–121 Hz for the 2 kg resonator and 87–120 Hz for the 6 kg resonator, whereas the measured intervals are approximately 90–106 Hz and 93–113 Hz, respectively, indicating generally good agreement. Under the 6 kg condition, the high-frequency attenuation band does not shift monotonically downward with increasing mass as in the idealized model; instead, it shifts slightly upward. This phenomenon can be attributed to the sensitivity of the higher-order torsional/rocking modes to the base plate–sand contact stiffness: the contact stiffness evolves with the resonator mass, and the real sand interface exhibits pronounced nonlinearity and discreteness. Consequently, the measured positions of the higher-order band-gaps deviate from the idealized simulation predictions. In contrast, the low-frequency attenuation band (BG2), dominated by the fundamental translational mode, is mainly controlled by the spring stiffness and is less affected by contact conditions; therefore, the measured low-frequency shift agrees well with the simulation trend.

In addition, a finite 7 × 6 periodic array cannot support a strict complete Bloch band-gap; the measured attenuation is essentially a combined result of local resonance, scattering, and soil damping. Quantitative deviations from the idealized infinite-periodic model are therefore expected, which accounts for the differences between the measured attenuation bands and the simulated band-gaps listed in Table 4. In particular, soil–resonator contact nonlinearity can shift band-gap boundaries and mainly causes quantitative deviations of the higher-order band-gaps (BG3 and BG4) from Bloch predictions, whereas BG2 retains a robust mass-driven frequency-shift trend. A detailed characterization of soil nonlinearity is left for future work. Separately, owing to the frequency limit of the vibration exciter and environmental noise, the present experiment cannot yet resolve a measurable ultra-low-frequency band-gap to verify BG1.

### 4.2. Attenuation of Separated Surface-Wave Components

**Figure 9:**
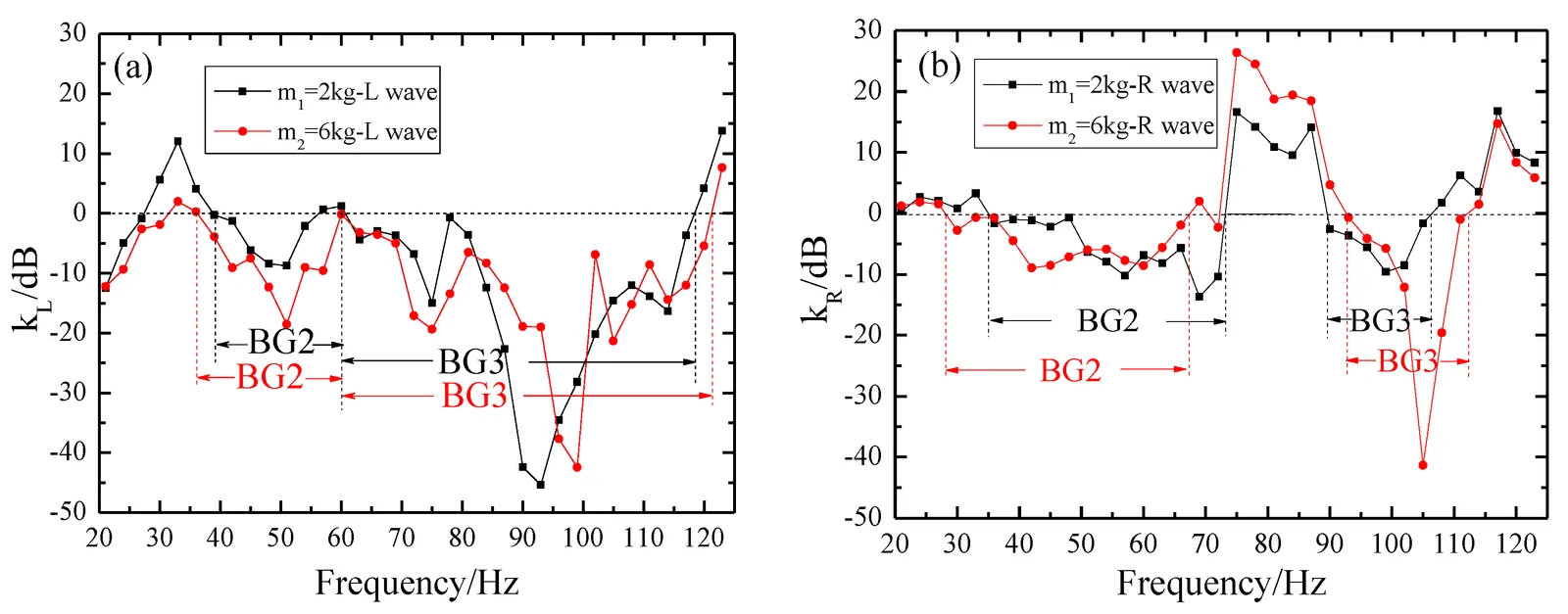
Energy attenuation for different wave types: (**a**) Love waves, (**b**) Rayleigh waves.

For Love waves (Figure 9a), BG2 of the 2 kg resonator spans 39–60 Hz. Increasing the mass to 6 kg lowers the lower bound to 36 Hz while the upper bound remains at approximately 60 Hz, broadening the bandwidth by 3 Hz. An ultra-broadband attenuation band (BG3), spanning 60–118 Hz for 2 kg and 60–121 Hz for 6 kg, is also observed, indicating excellent Love-wave suppression. This result is consistent with the prediction of the six-DOF model in Section 2: increasing the mass enlarges the mass matrix and moments of inertia, whereas the baseplate–sand contact stiffness increases only sublinearly with mass (Equation (3)). According to the Rayleigh quotient (Equation (4)), the frequencies of the rocking/shear–tilt modes shift downward overall. Since Love waves are dominated by horizontally polarized SH components, they can effectively excite in-plane rocking and shear–tilt modes but cannot excite the out-of-plane torsional mode; therefore, their high-frequency attenuation mainly originates from BG3. The downward shift in the lower bound of BG2 and the slight broadening of its bandwidth indicate that mass tuning offers practical tunability for low-frequency Love-wave suppression.

For Rayleigh waves (Figure 9b), BG2 shifts from 35–73 Hz (2 kg) to 28–67 Hz (6 kg), with the lower and upper bounds decreasing by 7 Hz and 6 Hz, respectively, while the bandwidth remains largely unchanged, consistent with the simulation results. This band is dominated by the rocking mode, whose generalized stiffness is mainly governed by the spring stiffness and is relatively less affected by contact-stiffness perturbations; hence, the measured downward shift agrees well with the mass-driven low-frequency shift predicted in Section 2. In the high-frequency Rayleigh band (BG3), the 2 kg and 6 kg cases exhibit intervals of 90–106 Hz and 93–113 Hz, respectively. Unlike Love waves, Rayleigh waves possess a vertical vibration component and can also excite the out-of-plane torsional mode. When the mass reaches 3 kg or above, higher-order modes decouple to form BG4; therefore, the high-frequency attenuation band at 6 kg may include a contribution from BG4. Higher-order band-gaps are highly sensitive to the baseplate–sand contact stiffness *k*_c_(*m*); the nonlinearity and discreteness of real sand cause the measured positions to deviate from the idealized infinite-period Bloch prediction, leading to the 6 kg high-frequency band not shifting monotonically downward as in the idealized model. 

Overall, the Love/Rayleigh decomposition results validate the theoretical predictions of the six-DOF model in Section 2 regarding mass-driven band-gap downshift and modal selectivity, and further indicate that the tuning of higher-order band-gaps in real sites must account for wave polarization and contact nonlinearity.

## 5. Conclusions

This study combines Bloch simulations with full-scale field tests on a 7 × 6 mass-tunable spring-resonator array to investigate band-gap tuning for surface waves. Key findings are: 

Mass-driven low-frequency shift and BG4 emergence: Increasing resonator mass (2 → 6 kg) shifts all band-gaps toward lower frequencies. When the mass reaches ≥3 kg, higher-order modes decouple, generating a fourth band-gap (BG4) in simulations. Experimentally, the total-energy attenuation band BG2 shifts from 48–74 Hz at 2 kg to 35–68 Hz at 6 kg, confirming this trend. Love/Rayleigh decomposition further shows that BG2 of Love waves shifts from 39–60 Hz to 36–60 Hz, while that of Rayleigh waves shifts from 35–73 Hz to 28–67 Hz.

Modal selectivity: BG2 and BG3 respond to both Love and Rayleigh waves. BG4, corresponding to an out-of-plane torsional mode, can only be excited by Rayleigh waves via their vertical component, not by Love waves. This polarization-dependent behavior implies that the additional attenuation band from BG4 is available only for Rayleigh-wave-dominated sites; Love-wave-dominated sites still rely primarily on BG2 and BG3. The high-frequency attenuation of Love waves mainly originates from BG3, whereas that of Rayleigh waves may include contributions from BG4.

The resonator-mass tuning approach offers a reversible, low-cost, and practically feasible strategy for in situ band-gap reconfiguration tailored to site-specific spectra. Overall, mass tuning effectively enables band-gap adaptation, and this full-scale dataset provides a valuable experimental reference for designing tunable seismic metasurfaces under realistic soil conditions.

## Figures and Tables

**Table 1:** Design parameters.

*a* (m)	*L*_1_ (m)	*L*_2_ (m)	*L*_3_ (m)	*H*_0_ (m)	*H*_1_ (m)	*H*_2_ (m)	*H*_3_ (m)
0.3	0.1	0.04	0.06	1.0	0.20	0.05	0.01

**Table 2:** Material parameters.

Material	Density *ρ* (kg/m^3^)	Young’s Modulus *E* (MPa)	Poisson’s Ratio *ν*
River sand	1400	20.0	0.47
Poplar wood	400	200	0.35
PET	1320	2.53 × 10^3^	0.35
Iron sand	4400	1.00 × 10^5^	0.31
Manganese steel	7850	2.05 × 10^5^	0.25

**Table 3:** Modal frequencies and band-gaps of the six-DOF model for different masses.

Modal Mode	2 kg (Hz)	6 kg (Hz)	Dominant DOF(s) in the Six-DOF Model	Band-Gap Opened
*f*_x_	8.8	7.7	horizontal translation *u_x_*/*u_y_*	none (near or below the lower edge of BG1)
*f*_m_	48.3	35.8	vertical translation *u_z_* coupled with rocking *θ_x_*/*θ_y_* (mixed mode)	BG1
*f*_r1_	75.1	66.9	rocking *θ_x_*/*θ_y_*	BG2
*f*_r2_	109.6	87.2	higher-order rocking/shear–tilt	BG3
*f*_r3_	(nearly degenerate with *f*_r2_)	103.7	out-of-plane torsion *θ_z_*	BG4 (emerging for m ≥ 3 kg)

**Table 4:** Band-gap variations with resonator mass.

Mass *m* (kg)	BG1 (Hz)	BG2 (Hz)	BG3 (Hz)	BG4 (Hz)
2	9.4–47.5	48.8–74.4	75.8–109.3	—
3	8.7–40.0	40.3–70.5	72.4–92.8	94.1–107.3
4	8.5–38.1	39.4–69.0	70.9–90.6	91.9–105.1
5	8.3–36.0	37.3–67.5	69.5–89.2	90.5–103.6
6	7.6–35.7	36.7–65.8	67.8–87.6	88.8–103.1

## Data Availability

The original contributions presented in this study are included in the article. Further inquiries can be directed to the corresponding authors.

## Abbreviations

The following abbreviations are used in this manuscript:

BG(1–4)	band-gap(1–4)
MTC	mass-tuning cup
six-DOF	six degrees of freedom
PET	Polyethylene terephthalate
